# Sitamaquine as a putative antileishmanial drug candidate: from the mechanism of action to the risk of drug resistance

**DOI:** 10.1051/parasite/2011182115

**Published:** 2011-05-15

**Authors:** P.M. Loiseau, S. Cojean, J. Schrével

**Affiliations:** 1 Groupe Chimiothérapie Antiparasitaire, UMR 8076 CNRS, Faculté de Pharmacie, Université Paris-Sud 11 92290 Châtenay-Malabry France; 2 Muséum National d’Histoire Naturelle, Département RDDM, UMR 7245 CNRS CP 52 61, rue Buffon 75231 Paris Cedex 05 France

**Keywords:** sitamaquine, leishmaniasis, aminoquinoline, drug action, sitamaquine, leishmaniose, aminoquinoléine, mécanisme d’action

## Abstract

Sitamaquine is a 8-aminoquinoline in development for the treatment of visceral leishmaniasis by oral route, no activity being observed on the experimental cutaneous leishmaniasis experimental models. Recent data explain how sitamaquine accumulate in *Leishmania* parasites, however its molecular targets remain to be identified. An advantage of sitamaquine is its short elimination half-life, preventing a rapid resistance emergence. The antileishmanial action of its metabolites is not known. The selection of a sitamaquine-resistant clone of *L. donovani* in laboratory and the phase II clinical trials pointing out some adverse effects such as methemoglobinemia and nephrotoxicity are considered for a further development decision.

## Abstract (fr)

La sitamaquine est une 8-aminoquinoléine en développement pour le traitement exclusif de la leishmaniose viscérale par voie orale, aucune activité n’ayant été observée sur les modèles expérimentaux de leishmaniose cutanée. Les données récentes de la littérature expliquent comment la sitamaquine s’accumule chez les leishmanies ; cependant, les cibles moléculaires de cette molécule restent à être identifiées. Un avantage de la sitamaquine réside dans sa demi-vie d’élimination courte, limitant l’émergence rapide de chimiorésistance. De même, l’action antileishmanienne de ses métabolites n’est pas encore connue. La selection d’un clone de *Leishmania donovani* résistant à la sitamaquine en laboratoire et les essais cliniques de phase II mettant en évidence des effets secondaires, tels que la methémoglobinémie et la néphrotoxicité, sont pris en considération pour une décision quant au développement de cette molécule.

## Introduction

Leishmaniases are tropical and sub-tropical diseases caused by the parasite protist belonging to the genus *Leishmania*. Two basic forms of leishmaniases occurs: i) visceral leishmaniasis (VL) or “Kala-azar” is caused by *Leishmania donovani* and *Leishmania infantum* (also known *L. chagasi* in South America), and ii) cutaneous leishmaniasis (CL) is caused by about 15 species of *Leishmania*, *L. tropica* (recidivan leishmaniasis) in the old world, and two possible forms in Latin America, diffuse CL (*L. guyanensis*, *L. amazonensis*) and mucocutaneous form with destruction of mouth mucosa, pharynx and facial tissue (*L. braziliensis*) (WHO, 2010). VL, the most severe form, is fatal without treatment. The leishmaniases are prevalent in about 88 countries: 350 million (M) people living in endemic aeras. The morbidity of about 12-14 M people and roughly 1.5-2 M new cases per year from whom 0.4-0.5 M for VL mainly in India, Nepal, Bengladesh, Brazil and Sudan (WHO, 2010). The global mortality is about 60,000 people (Desjeux, 2004). Conventional treatments include antimonial drugs (Glucantime^®^ and Pentostam^®^), amphotericin B and its liposomal formulation (AmBisome^®^) which are used by parenteral route. A phosphorylcholine ester of hexadecanol, designated as miltefosine, originally developed as an anticancer drug (Muschiol *et al.*, 1987) was shown to be the first oral drug against visceral (Jha *et al.*,1999, Sundar *et al.*, 2002) and cutaneous (Soto *et al.*, 2004; Sinderman & Engel, 2006, Soto & Toledo, 2007). However, it can be noticed that miltefosine (Impavido^®^) possesses a long half-life able to generate resistant *Leishmania* isolates and exhibits contraindication in pregnancy because of adverse effects (Jha *et al.*, 1999, Soto & Berman, 2006). Despite these limitations, miltefosine is now success-fully proposed in combination with AmBisome^®^ in order to prevent drug resistance to both the drugs (Sundar *et al.*, 2008).

**Fig 1.**
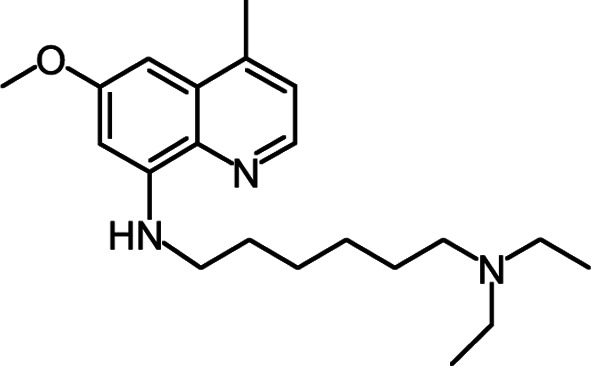
Sitamaquine (WR-6026), a 8-aminoquinoline analog.

Anyway, new compounds active by oral route should be developed in case of failure of this AmBisome^®^- miltefosine bitherapy in the future. Sitamaquine (WR- 6026) is a 8-aminoquinoline analog (Fig. 1) discovered by the Walter Reed Army Institute of Research (WRAIR, USA) and in development with GlaxoSmithKline (UK) for the oral treatment of VL. Sitamaquine was first synthesized as part of the collaborative antimalarial program in the US (1944-1950) that led to primaquine (Elderfield *et al.*, 1955). Sitamaquine is an orally active drug and appears as promising agent for treatment of VL both in India (Jha *et al.*, 2005) and Africa (Wasunna *et al.*, 2005).

## *In Vitro* and in *Vivo* Sitamaquine Activities on Leishmaniasis Models

Recent *in vitro* parasite evaluation confirmed the antileishmanial properties of sitamaquine dihydrochloride against a range of *Leishmania* species responsible for either cutaneous or visceral leishmaniasis, with ED50 values against amastigotes in a range from 2.9 to 19.0 microM (Garnier *et al.*, 2006). In fact, the antileishmanial activity of 8-aminoquinoline was revealed more than fifty years ago when a series of 6-methoxy-8-alkylpiperazinoalkylaminoquinoline derivatives were shown to exhibit both a higher activity than pentavalent antimonials and oral availability against *L. donovani* in the hamster model (Beveridge *et al.*, 1958). Later, a series of 4-methyl-6-methoxy-8-aminoquinolines called lepidines was shown to be several hundred times more active than pentavalent antimonials in a rodent model (Kinnamon *et al.*, 1978). Structure-activity relationships of methoxy- and hydroxy-substituted compounds were further investigated in a *L. tropica*-macrophage model *in vitro* (Berman & Lee, 1983). Among them, primaquine exhibited a noteable high activity and 8-[[6-(diethylamino) hexyl]amino]-6-methoxy-4-methylquinoline or WR6026, now called sitamaquine was 708 times more active than meglumine antimoniate (Glucantime^®^) against *L. donovani* in hamsters (Kinnamon *et al.*, 1978).

On *L. major* cutaneous lesions in BALB/c mice, different topical sitamaquine dihydrochloride formulations using topically acceptable excipients were evaluated *in vivo* without success since no reduction of the parasite burden and lesion progression was observed (Garnier *et al.*, 2006).

## Mechanism of Action

Although the sitamaquine effects on the parasite have been visualized via alterations in their morphology (Langreth *et al.*, 1983), the molecular targets of sitamaquine are still unknown. However, the sequential steps of interactions of sitamaquine with parasites are now well documented. Sitamaquine entry into *Leishmania* does not involve a transporter (López-Martín *et al.*, 2008). As a lipophilic weak base, the sitamaquine accumulation into *Leishmania* promastigotes occurs along an electrical gradient involving two steps: first, the positively charged sitamaquine interacts with the anionic polar head groups of membrane phospholipids, and second, the sitamaquine insertion into the parasite plasma membranes results of a subsequent hydrophobic interaction between acyl chains of phospholipids and the hydrophobic quinoline ring leading to a deeper insertion of the drug into the lipid monolayer (Dueñas-Romero *et al.*, 2007). This process is energy- and sterol-independent (Coïmbra *et al.*, 2010). However, this affinity of sitamaquine for membranes is transitory since the main sitamaquine location was found into the cytosol (Coïmbra *et al.*, 2010). In contrast, an energy-dependent efflux has been evidenced but the nature of the protein involved in this efflux remains to be elucidated (Coïmbra *et al.*, 2010). NMR study of motile lipids showed that sitamaquine does not affect lipid trafficking in *Leishmania* (Coïmbra *et al.*, 2010). Once internalized, sitamaquine rapidly accumulates into acidic compartments, mainly acidocalcisomes [acid vacuoles containing most of the cellular calcium] (López-Martín *et al.*, 2008). This accumulation in acidocalcisomes allows to their alkalization (Vercesi *et al.*, 2000). A rapid collapse of the mitochondrial innermembrane potential was also observed (Vercesi et al., 1992). However, the antileishmanial action of sitamaquine is not related to its level of accumulation in acidocalcisomes (López-Martín *et al.*, 2008). Proteomic analysis are running now to identify the sitamaquine targets.

## Bioavailability

Pharmacokinetics data in humans showed that sitamaquine has a short elimination half-life (about 26 hr) in contrast to miltefosine half-life (150-200 hr) (Theoharides *et al.*, 1987). The metabolism of sitamaquine was studied in a rat hepatic microsomal system (Theoharides *et al.*, 1985). Two metabolites were found: 8(-6-ethylaminohexylamino)- 6-methoxy-lepidine and 8(-6-diethylaminohexylamino)-6-methoxy-4-hydroxy-methyl-quinoline (Yeates, 2002). The formation of both metabolites was NADPH-dependent. The formation of both metabolites seems to be catalyzed by different cytochrome P450 isozymes. No more data are so far available to understand the importance of metabolites in the sitamaquine action.

## Clinical Trials on Visceral Leishmaniasis

First phase II assays performed in Kenya on 16 patients were encouraging enough to be continued further (Sherwood *et al.*, 1994). In phase II assays in India with 120 VL patients (Jha *et al.*, 2005), and in Kenya with 95 VL patients (Wasunna *et al.*, 2005), sitamaquine was well tolerated with the doses ranging from 1.5 to 3 mg/mg/day, with vomiting and abdominal pains (about 10%), headache (also about 10%). Cyanosis (3%) in India was reported to be due to methemoglobinemia, a recognized side effect of 8-aminoquinolines for individuals with glucose-6-phosphate deshydrogenase (G6PD) deficiency (Jha *et al.*, 2005). Methemoglobinemia was not reported in the Kenyan study (Wasunna *et al.*, 2005). Renal adverse effects (nephritic syndrome 3% and glomerulonephritis 2% in India) were observed only for doses ≥ 2.5 mg /mg/day (Jha *et al.*, 2005), but effects on kidney need further investigation.

Another phase II clinical trial including dose-escalating safety and efficacy study was carried out in *L. chagasi* infected patients in Brazil (Dietze *et al.*, 2001). Cure rates were not successful since a lack of increased efficacy was observed with increased dosing above 2 mg/kg/day × 28. Nephrotoxicity was observed at 2.5 mg/kg/day in two patients and in the single patient administered 3.25 mg/kg/day (Dietze *et al.*, 2001).

On cutaneous leishmaniasis, because of the lack of activity on the *in vivo* models, no clinical development was performed with sitamaquine (Garnier *et al.*, 2006).

## Risk of Drug Resistance

The short elimination half-life of sitamaquine in mammals is in favour of a low probability of resistance emergence. However, in order to evaluate the risk of sitamaquine resistance in the field, a *L. donovani* promastigote line resistant to 160 μM sitamaquine was selected by *in vitro* drug pressure and some charachetristics of this resistant line were studied (Bories *et al.*, 2008). The resistant line was infective for murine peritoneal macrophages *in vitro* as its parent wild-type line but less infective for Balb/C mice, suggesting that a low transmission of resistant parasites could occur in the field. The sitamaquine IC_50_ on the resistant line was about five and three times higher than those of the wild-type line on promastigote and intramacrophage amastigote forms, respectively. No cross-resistance with other antileishmanial agents was observed, allowing to use another antileishmanial drug in case of sitamaquine resistance. However, this resistance was stable when parasites were subcultured in drug-free medium for a long time or after *in vivo* passage, suggesting that a maintenance at a constant level in the parasite populations. These considerations, apparently speculative, could be indicative from an epidemiological point of view.

## Conclusion

Few chemical series reach the clinical development in the field of leishmaniasis because the antileishmanial screening and toxicity bottlenecks are selective. Sitamaquine is the second orally active antileishmanial drug after miltefosine. The development of sitamaquine is slow because time is needed to ensure the safety of the drug, mainly at the level of methemoglobinemia and nephrotoxicity. Recent data show that resistance is at risk. However, the level of resistance obtained by *in vitro* drug pressure corresponds to a loss of susceptibility of 5-fold, that would be compatible with higher dosages if sitamaquine was not toxic. The major advantages of sitamaquine are its administration route and pharmacokinetics characteristics. Thus, its bioavailability is better than those of miltefosine. From all these data gathered in Table I, it is now probable that GSK company, the developer, will take a decision concerning the marketing of sitamaquine in the next future.

**Table I:** Physico-chemical and biological properties of sitamaquine, an antileishmanial agent active against visceral leishmaniasis.

Physico-chemical and biological properties of sitamaquine	
Chemical formula	C_21_H_33_N_3_O.2HCl (dihydrochloride)

Physical properties	Octanol/water partition coefficient: LogP = 5.84

	Molecular weight: 342.51 g 415.43 g (dihydrochloride)

Solubility	Dihydrochloride: water soluble (> 100 mg/ml at 25 °C)
	Ethanol soluble

Chemical characteristics	Weak base pKa = 4.2 (quinoline nitrogen) 10.4 (amine side chain)

Behaviour in biological fluids	Affinity for proteins (Duenas et al., 2007)

Interaction with host cell / parasite	Affinity for negative phospholipids (Duenas et al., 2007)
	Morphology alteration of *Leishmania* (Langreth *et al.*, 1983)

Uptake and accumulation in	Electrical gradient diffusion (Duenas et al., 2007)

*Leishmania donovani* promastigotes	No affinity for sterols (Soares *et al.*, 2010)
	No transporter suspected (López-Martín *et al.*, 2008)
	Energy dependent efflux (Soares *et al.*, 2010)

Intracellular targets	Accumulation in acidocalcisomes (Vercesi *et al.*, 2000)
	Rapid collapse of the mitochondrial inner-membrane potential (Vercesi *et al.*, 2002)
	Sitamaquine susceptibility not related to accumulation into acidocalcisomes (López-Martín *et al.*, 2008)

Bioavailability	Plasma half-life: 26.1 hr
	4-CH_2_OH as major urinary metabolite (Theoharides *et al.*, 1987)

Clinical trials Phase II	High efficacy rate at doses 1.5-3 mg/kg/day × 28 by oral route
	Trials in India (Jha *et al.*, 2005)
	Trials in kenya (Wasunna *et al.*, 2005)

Toxicity / adverse effects (% of patients)	Vomiting Abdominal pains Headache (10 %) Methemoglobinemia (3 %) Cyanosis (3 %) Renal adverse effects: if doses > 2.5 mg/kg Nephritic syndrome (3 %) Glomerulonephritis (2 %)	(Jha *et al.*, 2005)
	No methemoglobinemia in Kenya (Wasunna *et al.*, 2005)

Resistance	At risk: obtained *in vitro* (Bories *et al.*, 2008)

## References

[R1] Berman J.D. & Lee L.S. Activity of 8-aminoquinolines against *Leishmania tropica* within human macrophages *in vitro*. American Journal of Tropical Medicine and Hygiene, 1983, 32, 753–759 6881421 10.4269/ajtmh.1983.32.753

[R2] Beveridge E., Goodwin L.G. & Walls L.P. A new series of leishmanicides. Nature, 1958, 182, 316–317 13577825 10.1038/182316b0

[R3] Bories C., Cojean S., Huteau F. & Loiseau P.M. Selection and phenotype characterisation of sitamaquine-resistant promastigotes of *Leishmania donovani*. Biomedicine and Pharmacotherapy, 2008, 62, 164–167 10.1016/j.biopha.2007.12.006 18249083

[R4] Coïmbra E.S., Libong D., Cojean S., Saint-Pierre-Chazalet M., Solgadi A., Le Moyec L., Duenas-Romero A.M., Chaminade P. & Loiseau P.M. Mechanism of interaction of sitamaquine with *Leishmania donovani*. The Journal of Antimicrobial Chemotherapy, 2010, 65, 2548–2555 20956354 10.1093/jac/dkq371

[R5] Desjeux P. Leishmaniasis: current situation and new perspectives. Comparative Immunology, Microbiology and Infectious Diseases, 2004, 27, 305–318 10.1016/j.cimid.2004.03.004 15225981

[R6] Dietze R., Carvalho S.F.G., Valli L.C., Berman J., Brewer T., Milhous W., Sanchez J., Schuster B. & Grogl M. Phase II trial of WR6026, an orally administrated 8-aminoquinoline, in the treatment of visceral leishmaniasis caused by *Leishmania chagasi*. American Journal of Tropical Medicine and Hygiene, 2001, 65, 685–689 11791957 10.4269/ajtmh.2001.65.685

[R7] Dueñas-Romero A.M., Loiseau P.M. & Saint-Pierre-Chazalet M. Interaction of sitamaquine with membrane lipids of *Leishmania donovani* promastigotes. Biochimica et Biophysica Acta, 2007, 1768, 246–252 16945323 10.1016/j.bbamem.2006.07.003

[R8] Elderfield R.C., Mertel H.E., Mitch R.T., Wempen I.M. & Werble E.J.. Synthesis of primaquine and certain of its analogs. Journal of the American Chemical Society, 1955, 77, 4816–4819

[R9] Garnier T., Brown M.B., Lawrence M.J. & Croft S.L. *In vitro* and *in vivo* studies on a topical formulation of sitamaquine dihydrochloride for cutaneous leishmaniasis. Journal of Pharmacy and Pharmacology, 2006, 58, 1043–1054 16872550 10.1211/jpp.58.8.0004

[R10] Jha T.K., Sundar S., Thakur C.P., Bachmann P., Karbwang J., Fischer C., Voss A. & Berman J. Miltefosine, an oral agent, for the treatment of Indian visceral leishmaniasis. New England Journal of Medicine, 1999, 341, 1795–800 10588964 10.1056/NEJM199912093412403

[R11] Jha T.K., Sundar S., Thakur C.P., Felton J.M., Sabin A.J. & Horton J. A phase II dose-ranging study of sitamaquine for treatment of visceral leishmaniasis in India. American Journal of Tropical Medicine and Hygiene, 2005, 73, 1005–1011 16354802

[R12] Kinnamon K.E., Steck E.A., Loizeaux P.S., Hanson W.L., Chapman W.L. Jr. & Waits V.B. The antileishmanial activity of lepidines. American Journal of Tropical Medicine and Hygiene,, 1978, 27, 751–757 686239 10.4269/ajtmh.1978.27.751

[R13] Langreth S.G., Berman J.D., Riordan G.P. & Lee L.S. Finestructure alterations in *Leishmania tropica* within macrophages exposed to antileishmanial drugs *in vitro*. Journal of Protozoology, 1983, 30, 555–561 6315928 10.1111/j.1550-7408.1983.tb01421.x

[R14] López-Martín C., Pérez-Victoria J.M., Carvalho L., Castanys S. & Gamarro F. Sitamaquine sensitivity in *Leishmania* species is not mediated by drug accumulation in acidocalcisomes. Antimicrobial Agents and Chemotherapy, 2008, 52, 4030–4036 18794384 10.1128/AAC.00964-08 PMC2573096

[R15] Muschiol C., Berger M.R., Schuler B., Scherf H.R., Garzon F.T., Zeller W.J., Unger C., Eibl H.J. & Schmähl D. Alkyl phosphocholines: toxicity and anticancer properties. Lipids, 1987, 22, 930–934 3444388 10.1007/BF02535558

[R16] Sherwood J.A., Gachihi G.S., Muigai R.K., Skillman D.R., Mugo M., Rashid J.R., Wasunna K.M., Were J.B., Kasili S.K. & Mbugua J.M. et al. Phase II efficacy trial of an oral 8-aminoquinoline (WR6026) for treatment of visceral leishmaniasis. Clinical Infectious Diseases, 1994, 19, 1034–1039 7888530 10.1093/clinids/19.6.1034

[R17] Sindermann H. & Engel J. Development of miltefosine as an oral treatment for leishmaniasis. Transactions of the Royal Society of Tropical Medicine and Hygiene, 2006, 100(Suppl 1), S17–S20 16730362 10.1016/j.trstmh.2006.02.010

[R18] Soto J., Arana B.A., Toledo J., Rizzo N., Vega J.C., Diaz A., Luz M., Gutierrez P., Arboleda M., Berman J.D., Junge K., Engel J. & Sindermann H. Miltefosine for new world cutaneous leishmaniasis. Clinical Infectious Diseases, 2004, 38, 1266–1272 15127339 10.1086/383321

[R19] Soto J. & Berman J. Treatment of New World cutaneous leishmaniasis with miltefosine. Transactions of the Royal Society of Tropical Medicine and Hygiene, 2006, 100(Suppl 1), S34–S40 16930649 10.1016/j.trstmh.2006.02.022

[R20] Soto J. & Toledo J.T. Oral miltefosine to treat new world cutaneous leishmaniasis. Lancet Infectious Diseases, 2007, 7, 7 17182338 10.1016/S1473-3099(06)70665-X

[R21] Sundar S., Jha T.K., Thakur C.P., Engel J., Sindermann H., Fischer C., Junge K., Bryceson A. & Berman J. Oral miltefosine for Indian visceral leishmaniasis. New England Journal of Medicine, 2002, 347, 1739–1746 12456849 10.1056/NEJMoa021556

[R22] Sundar S., Rai M., Chakravarty J., Agarwal D., Agrawal N., Vaillant M., Olliaro P. & Murray H.W. New treatment approach in Indian visceral leishmaniasis: single-dose liposomal amphotericin B followed by short-course oral miltefosine. Clinical Infectious Diseases, 2008, 47, 1000–1006 18781879 10.1086/591972

[R23] Theoharides A.D., Chung H., Velazquez H. Metabolism of a potential 8-aminoquinoline antileishmanial drug in rat liver microsomes. Biochemical Pharmacology, 1985, 34, 181–188 3917669 10.1016/0006-2952(85)90122-4

[R24] Theoharides A.D., Kim M.M., Ashmore R.W. & Shipley L.A. Identification and quantification of human urinary metabolites of a candidate 8-aminoquinoline antileishmanial drug WR-6026. Proceedings of American Society of Experimental Biology, 1987, 46, 865

[R25] Vercesi A. & Docampo R. Ca^2+^ transport by digitonin-permeabilized *Leishmania donovani*. Effects of Ca^2+^, pentamidine and WR-6026 on mitochondrial membrane potential in situ. Biochemical Journal, 1992, 284, 463–467 1376113 10.1042/bj2840463 PMC1132661

[R26] Vercesi A., Rodrigues C., Castisti R. & Docampo R. Presence of a Na^(+)^/H^(+)^ exchanger in acidocalcisomes of *Leishmania donovani* and their alkalization by anti-leishmanial drugs. FEBS Letters, 2000, 473, 203–206 10812075 10.1016/s0014-5793(00)01531-3

[R27] Wasunna M.K., Rhashid J.R., Mbui J., Kirigi G., Kinoti D., Lodenyo H., Felton J.M., Sabin A.J. & Horton J. A phase II dose-increasing study of sitamaquine for treatment of visceral leishmaniasis in Kenya. American Journal of Tropical Medicine and Hygiene, 2005, 73, 871–876 16282296

[R28] World Health Organization Leishmaniasis Disease Burden. Available from: http://www.who.int/leishmaniasis, 2010

[R29] Yeates C. Sitamaquine (GlaxoSmithKline/Walter Reed Army Institute). Current Opinion in Investigational Drugs, 2002, 3, 1446–1452 12431016

